# Home confinement’s impact on myopia control by using orthokeratology in school-aged children

**DOI:** 10.1186/s12886-023-02969-3

**Published:** 2023-06-05

**Authors:** Boyuan Zhang, Hongfei Liao, Fen Xiong, Tian Mao, Lili Wu, Yue Li, Chao Xiong

**Affiliations:** 1 Department of Orbital Diseases, Affiliated Eye Hospital of Nanchang University, Nanchang, 330006 China; 2 Academic of Optometry, Nanchang University, Nanchang, China

**Keywords:** Home confinement, Orthokeratology, Myopia control, School-aged children

## Abstract

**Background:**

Home confinement during the epidemic has a significant impact on the lifestyle and behavior of school-aged children, who have exhibited an increase in the prevalence and development of myopia. Our research will look at if home confinement will affect school-aged children on myopia control with orthokeratology.

**Method:**

Data on axial length was gathered from school-aged children who had received OK lenses treatment. The entire data was separated into subgroups based on gender, age, and initial refraction, and the AL changes for each period were calculated using the formula defined in our study. Finally, the acquired data will be examined using various statistical approaches, and the ideas of slow, moderate, and rapid myopia progression will be applied to our study.

**Result:**

A total of 258 study subjects met the requirements to be included in the study. We discovered that the percentage of rapid myopia growth increased during the epidemic. In addition, the AL changes before and during the epidemic were found to be statistically significant in 171 subjects in the overall data. (*P* = 0.041) In the high age group, the AL changes before and during the epidemic、(*P* = 0.033) before and after the epidemic (*P* = 0.023) were found to be statistically significant. The AL changes before and during the epidemic (*P* = 0.035) were shown to be statistically significant in the moderate myopia group. Finally, we did not find statistically significant results for other groups.

**Conclusion:**

We cannot conclude that home confinement did have a negative impact on myopia control with orthokeratology in school-aged children. But we found there was an increase in the percentage of patients with OK treatment that had fast myopia progression during the confinement. We also observed that older children with higher initial refraction were more likely to be affected by home confinement.

**Supplementary Information:**

The online version contains supplementary material available at 10.1186/s12886-023-02969-3

## Background

Myopia has been more common in recent years, such as in the United States, [1] Western Europe, [2–4] and particularly in some East and Southeast Asian countries [5]. We also need to know that as low myopia develops into high myopia, the risk of ocular diseases such as glaucoma, [6–9] cataracts, [10–13] retinal detachment and atrophy [14–18] increases significantly, all of which can result in vision loss. Myopia causes great challenges for people. Nevertheless, most people still regard myopia as a benign condition because good vision can be obtained with glasses, contact lenses, and refractive surgery, [19] and it has been well documented that orthokeratology control myopia better than glasses in children and adolescents [20]. These phenomena suggest that orthokeratology plays an important role in our life.

The 2020 neocon epidemic ravaging the world is not only a public health emergency but also a global threat. As an emergency measure, the government has ordered citizens to stay home and suspended schools to prevent the further spread of the infection. As of March 26, 2020, 150 million children and adolescents in 165 countries are affected by the closures [21]. In response to the 2019 outbreak of coronavirus disease (neo-coronavirus pneumonia), the Chinese government has ordered nationwide school closures as an emergency measure to prevent the spread of the infection. Public activities are not encouraged. The Chinese Ministry of Education estimates that more than 220 million children and adolescents are confined to their homes; this includes 180 million primary and secondary school students and 47 million preschoolers. Due to China's strong management system, emergency family education programs are strictly enforced [22]. We must note that the lifestyle behaviors of children and adolescents, such as physical activity (PA) and sedentary behavior (SB), may have been greatly affected by the prolonged school closures and home confinement during the epidemic [21]. It is well known that both reduced PA and prolonged SB are associated with negative physical and mental health [23]. The occurrence and progression of myopia in children and adolescents are substantially elevated during COVID-19 home confinement [24–26].

However, studies on the use of orthokeratology for myopia control during COVID-19 home confinement remain little explored. Our study aims to investigate the effect of COVID-19 home confinement on myopia control with orthokeratology by using multiple statistical tests. Also, data on the causes of visual impairment and blindness are important for the development of public health policies, but a comprehensive analysis of changes in prevalence over time is lacking [27]. This paper will, to some extent, provide some basis and insight for the development of relevant public health policies in the future.

## Method

Observational research with a stratified, parallel-group design was used to determine if home confinement affects school-aged children's myopia control with orthokeratology. We collected the AL date of myopic school-aged children treated with orthokeratology before home confinement, and we phone these patients after home confinement, instructing them to attend the routine review at the hospital. All research participants were split into low and high age groups based on whether they were above the age of 12, low and moderate myopia groups based on whether their initial refraction was greater than -3.0 D, and male and female groups based on gender. The AL change for each time was calculated using the formula stated. Statistical analysis was used to determine if home confinement had an impact on the myopia control with orthokeratology in the total data and each subgroup.

In addition, slow myopia growth was defined as an increase of the AL less than 0.09 mm every six months (i.e., 0.18 mm per year), moderate myopia growth as an increase of the AL more than 0.09 mm, and no more than 0.18 mm every six months, and rapid myopia growth as an increase of the AL more than 0.18 mm every six months (i.e., 0.36 mm per year) [28]. The percentages of slow myopic growth, moderate myopic growth, and rapid myopic growth were calculated for the total data and each subgroup before, during, and after the epidemic. This method will in another way test whether the impact of home confinement during the epidemic exists, together with the statistical analysis to enhance the credibility of the study.

## Subjects

Myopic school-aged children aged 8–17 years who had undergone orthokeratology treatment at the Eye Hospital of Nanchang University before the home confinement was selected. The data relating to the experiment were reorganized, and the subjects were strictly screened according to the inclusion and exclusion criteria: (1) Inclusion criteria: myopic school-aged children with initial refraction among -0.5D to -5.0D; myopic school-aged children who are reviewed at least 3 times with a certain interval for each review. (2) Exclusion criteria: patients with strabismus, amblyopia, and other eye diseases; patients using atropine in combination therapy; patients with high myopia corrected with orthokeratology in combination with frame glasses (Table 1).

**Table 1:** Inclusion and exclusion criteria

Inclusion criteria	Exclusion criteria
• 8 to 17 years old	• Have strabismus, amblyopia, or other eye diseases
• initial refraction among -0.5D to -5.0D	• Patients with high myopia treated with orthokeratology lens in combination with frame glasses
• At least three follow-up visits with a certain interval	• patients using atropine in combination therapy

The study followed the Declaration of Helsinki, and all enrolled patients and guardians signed an informed consent form.

## Evaluation index of myopia control

So many studies have shown a strong correlation between the AL and its refraction [29–32].  As indicated by the significant link observed between changes in refractive error (i.e., myopia progression) and changes in AL (i.e., axial growth of the eye), myopia development and progression are usually caused by excessive axial elongation of the eye [33–35]. Alignment-fitted gas permeable contact lenses can flatten the cornea, contaminating the lens's apparent influence on refractive error [36].  For these reasons, assessments of an individual's change in axial length are frequently used as the major outcome measure of myopia clinical studies in the myopia research field. In addition, myopic patients with orthokeratology are sometimes tested with a shorter AL, even if the measurement is repeated several times because the AL can change for a variety of reasons. For example, choroidal thickness is known to change throughout the day with a magnitude of up to 30 mm, [37, 38] and changes in choroidal thickness can also affect the measurement of the AL. There may be other unknown factors that can have an influence, so the AL growth of negative values should also be taken into account. Ultimately, the AL change will be used as the primary outcome in our study, rather than the AL growth or the change in refraction. The AL was measured by an IOL master (IOL Master; Zeiss Humphrey, Dublin, CA) and trained professionals. The patient underwent ocular AL measurement every time for routine review.

## Axial length change calculation

Since the time of each review was well recorded for the patients, we discovered that the patients' review times were not strictly as recommended, resulting in the interval between two reviews not being the same for each period, which ultimately made comparison impossible. In our study, the monthly AL change will be calculated uniformly, but because the monthly AL change is too small for comparison, and considering the home confinement’s time of nearly four months, we finally decided to compare the six-month AL change. The calculation formula is as follows.

$$\mathrm{Six}-\mathrm{month AL change}\hspace{0.17em}=\hspace{0.17em}((\mathrm{AL}2-\mathrm{AL}1)/\mathrm{interval months})\hspace{0.17em}\times \hspace{0.17em}6$$

If a patient is reviewed strictly as recommended over six months, three times the AL data will be recorded, and for the accuracy of the study results, the AL recorded at the review time point closer to the six-month anterior boundary will be chosen as AL1 and the AL recorded at the review time point closer to the six-month posterior boundary will be chosen as AL2. For example, if the patient had routine reviews on June 1, 2019, September 1, 2019, and December 1, 2019, we will select the AL data recorded on June 1, 2019, as AL1 and the AL data recorded on December 1, 2019, as AL2; if the patient has only two reviews in six months, the one with the earlier recorded at the time of the review of the two AL data will be used as AL1 and the other as AL2; if the patient had only 1 review in half a year, for those occurring before the epidemic, we would select the AL data recorded at an earlier time as AL1, the other as AL2, and for those occurring after the epidemic, we would select the AL data recorded at a later time as AL2 and the other as AL1. For the selection of AL1 and AL2 during the epidemic, we insisted on selecting the AL recorded during the two review times that spanned the end of January to the beginning of May, because the Nanchang students were undergoing home quarantine during this period of the epidemic.

Each review time record style for the month/day/year in our study. we define the review month to be represented by a number to facilitate the subsequent calculation of the review interval month, January represents the number 1, February represents the number 2, and so on, until December represents the number 12. Then we find that the calculation of the interval month will produce a systematic error if we just subtract the review month of AL1 from the review month of AL2. For example, the interval between the beginning of January and the end of May is about 5 months, and the interval between the end of January and the beginning of May is about 3 months, but the simple subtraction of the two months results in 4 months, and the absolute value of the AL change obtained from the former calculation increases compared with the actual one (because the interval month changes from 5 to 4 months), and the absolute value of the eye axial length change obtained from the latter calculation becomes smaller compared with the actual one (because the interval month changes from 3 to 4 months). Therefore, the inaccuracy of the interval month will have a direct effect on the calculation of the AL change, and this error may lead to the final failure to obtain the desired study results. To minimize this error, our method of calculating the interval time is introduced as follows:

$$\mathrm{When }30 \ge \mathrm{ AL}{2}^{\mathrm{^{\prime}}}\mathrm{s day}-\mathrm{AL}{1}^{\mathrm{^{\prime}}}\mathrm{s day}>20,\mathrm{ the interval month}=\mathrm{AL}{2}^{\mathrm{^{\prime}}}\mathrm{s review month}-\mathrm{AL}{1}^{\mathrm{^{\prime}}}\mathrm{s review month}+1\mathrm{ month}$$

$$\mathrm{When }20 \ge \mathrm{ AL}{2}^{\mathrm{^{\prime}}}\mathrm{s day}-\mathrm{AL}{1}^{\mathrm{^{\prime}}}\mathrm{s day }\ge 10,\mathrm{ the interval month}=\mathrm{AL}{2}^{\mathrm{^{\prime}}}\mathrm{s review month}-\mathrm{AL}{1}^{\mathrm{^{\prime}}}\mathrm{s review month}+0.5\mathrm{ months}$$

$$\mathrm{When }10>\mathrm{AL}{2}^{\mathrm{^{\prime}}}\mathrm{s day}-\mathrm{AL}{1}^{\mathrm{^{\prime}}}\mathrm{s day}>-10,\mathrm{ the interval month}=\mathrm{AL}2\mathrm{^{\prime}}\mathrm{s review month}-\mathrm{AL}1\mathrm{^{\prime}}\mathrm{s review month}$$

$$\mathrm{When }-10 \ge \mathrm{ AL}{2}^{\mathrm{^{\prime}}}\mathrm{s day}-\mathrm{AL}{1}^{\mathrm{^{\prime}}}\mathrm{s day }\ge -20,\mathrm{ the interval month}=\mathrm{AL}2\mathrm{^{\prime}}\mathrm{s review month}-\mathrm{AL}1\mathrm{^{\prime}}\mathrm{s review month}-0.5\mathrm{ months}$$

$$\mathrm{When }-20>\mathrm{AL}{2}^{\mathrm{^{\prime}}}\mathrm{s day}-\mathrm{AL}{1}^{\mathrm{^{\prime}}}\mathrm{s day }\ge -30,\mathrm{ the interval month }=\mathrm{AL}{2}^{\mathrm{^{\prime}}}\mathrm{s review month}-\mathrm{AL}{1}^{\mathrm{^{\prime}}}\mathrm{s review month}-1\mathrm{ month}$$

AL's day represents the value in the middle of the time record month/day/year, and the AL's review month is the value corresponding to its month. For example, when the time record is August 30 (th), 2019, the AL's day is 30, and the AL's review month is 8; when AL1's review time is May 30, 2019, and AL2's review time is August 30, 2019, AL2's day—AL1's day = 30–30 = 0 and the interval month = AL2's review month—AL1's review month—1 month = 8–5 = 3. Another thing to note is that when AL2's review time is the second year compared to AL1's review time, the interval month should be calculated by adding 12. For example, when AL1's review time is December 30, 2019 and AL2's review time is March 30, 2020, AL2's day—AL1's day = 30–30 = 0, interval month = AL2's review month + 12—AL1's review Month = 3 + 12—12 = 3. The AL change in our study was strictly calculated by a dedicated person according to the same requirements.

This calculation method will also produce some systematic errors, but it is acceptable for our study. Although it is possible to use software to accurately calculate daily growth, the authority of the software cannot be proven, and this method may be a better choice for countries where computers are not well-developed. The method used in our study not only reduces the systematic error, but the calculation is not much larger than the simple subtraction of the month between two reviews, which is worthwhile for improving the accuracy of the study.

## Statistical analysis

Data from the right eyes of all subjects who met the inclusion criteria were used for analysis, and for patients with monocular orthokeratology lenses, data from the eye with the orthokeratology were used. The statistical analyses were performed by dedicated personnel (SPSS software ver. 26.0; SPSS Inc., Chicago, IL), which considered *P* < 0.05 to be statistically significant. Based on age, gender, and initial refraction, the data was separated into two groups: low age and high age, male and female, low myopia and moderate myopia. Subjects in the low age group were 8–12 years old and in the high age group were 13–17 years old; subjects in the low myopia group had initial refraction among -0.5D to -3.0D and in the moderate myopia group had initial refraction among -3.25D to -5D. Each group was tested for normality, and one-way ANOVA was performed for data that met the normality distribution, and the Kruskal–Wallis H test was performed for data that did not meet the normality distribution. Due to the lack of randomization and the small sample size, we will use multiple statistical tests to detect the inherent regularity of the AL change during the epidemic. The difference between the AL change for each group before and during the epidemic, during and after the epidemic, before and after the epidemic will be tested for normality. Paired t-tests will be performed for groups ‘difference that meet normality distribution, and Wilcoxon signed-rank tests will be performed for groups’ difference that does not meet normality distribution. Finally, we will perform Repeated-measures ANOVA, a statistical method that is theoretically most appropriate for this study, on the total data and each subgroup as a supplement.

## Result

**Fig. 1:**
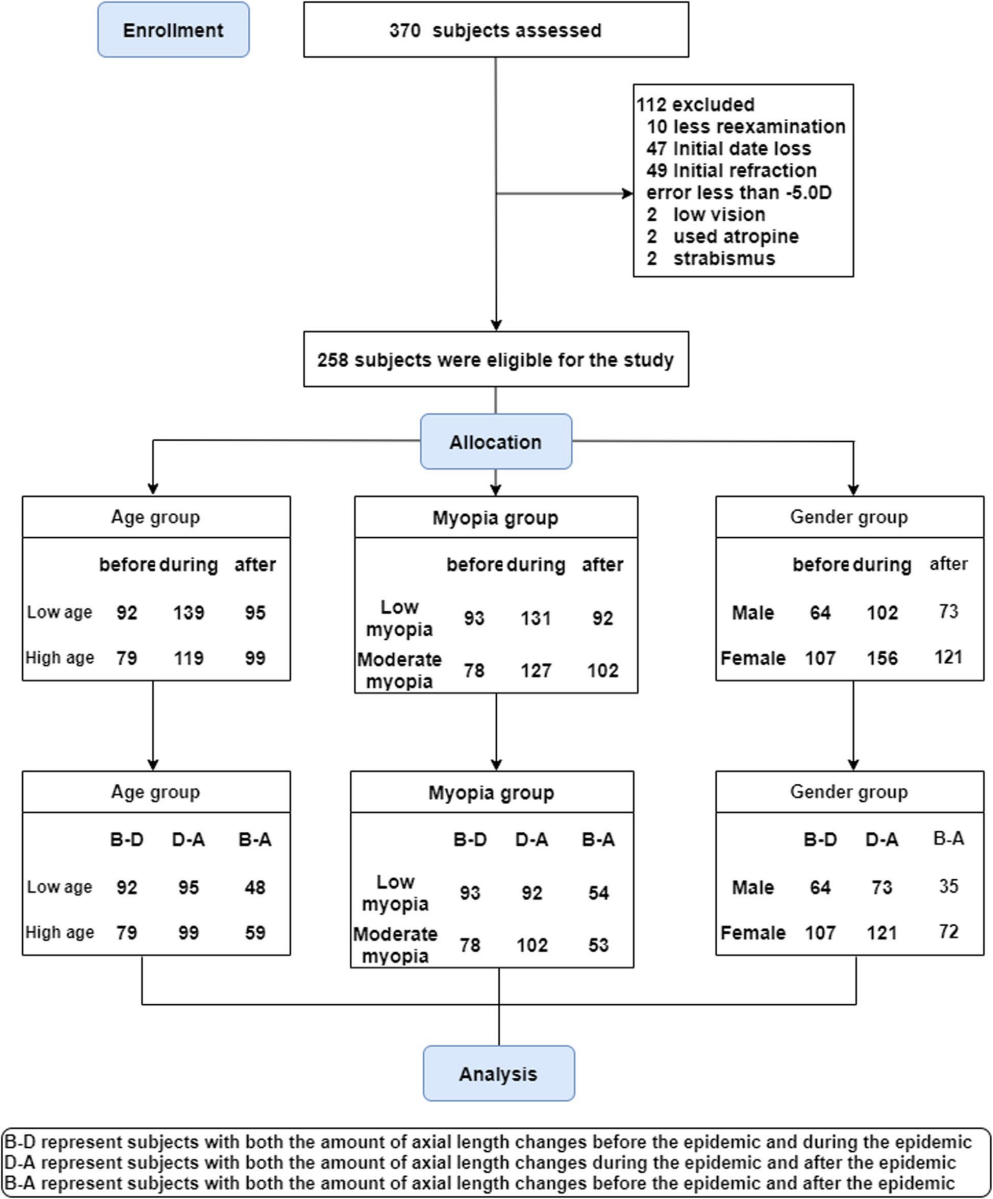
Flow chart

**Fig. 2:**
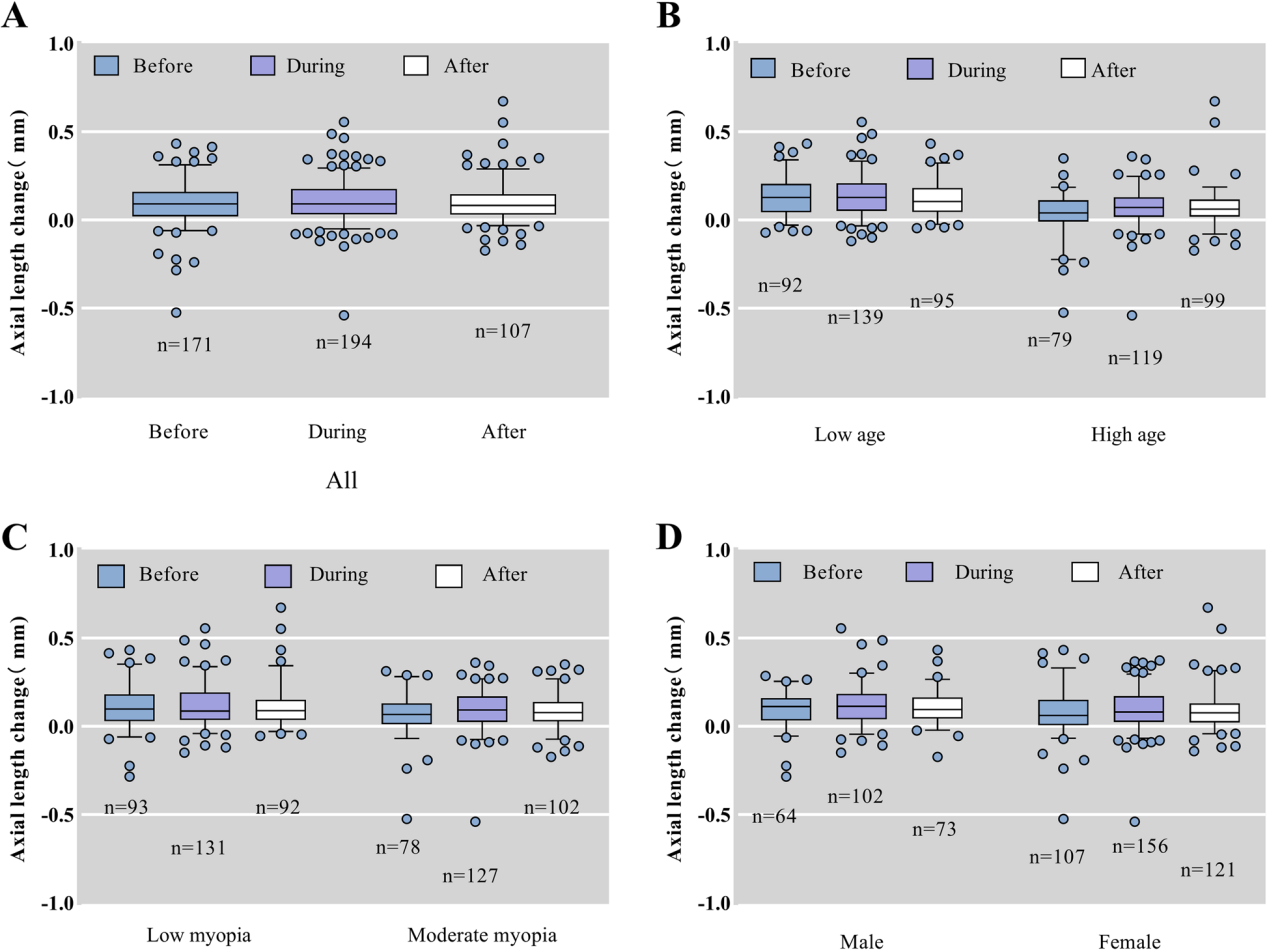
Box-scatter plot

In all, 370 subjects passed the phone screening and 258 (102 males and 156 females) subjects met the requirements to be included in the experiment, with a mean age of 12.34 ± 1.953 years, mean initial refraction of -2.956 ± 1.132 D (Table 2). Forty-nine subjects were excluded at the beginning stage because their initial refraction greater than -5.0 D, 47 subjects did not have initial AL data because they wanted to keep their AL measurement reports and eventually lost them, 10 subjects were followed up by telephone but did not eventually come to the hospital for a review, and 6 subjects (2 amblyopes, 2 strabismus, 2 atropine users) were excluded due to various special conditions. (Fig. 1) The mean ± SD AL change before the epidemic was 0.091 ± 0.126 mm in 171 subjects, during the epidemic was 0.105 ± 0.116 mm in 258 subjects, and after the epidemic was 0.095 ± 0.107 mm in 194 subjects. The Kruskal–Wallis H test for the AL change before, during, and after the epidemic revealed that *P* > 0.05, which was not statistically significant (Table 2). To rule out the possibility that this result was due to the intrinsic interaction of gender, age, and initial refraction, we analyzed each subgroup using the Kruskal–Wallis H test, and no statistical significance was found (Fig. 2). The basic parameters and statistical results are summarized in Table 2, respectively.

**Table 2:** Demographic Data (Mean ± SD) of each group

Group	Period	Age, y	Sex	Myopia, D	AL change, mm	N	Normality test	P
	Before		M = 102		0.091 ± 0.126	Nb = 171	0.000	
All	During	12.34 ± 1.953	FM = 156	-2.956 ± 1.132	0.105 ± 0.116	Nd = 258	0.280	0.364 > 0.05
	After		N = 258		0.095 ± 0.107	Na = 194	0.000	
	Before		M = 57		0.137 ± 0.112	Nb = 92	0.081	
Low age	During	10.86 ± 1.107	FM = 82	-2.781 ± 1.037	0.136 ± 0.117	Nd = 139	0.011	0.466 > 0.05
	After		N = 109		0.119 ± 0.098	Na = 95	0.007	
	Before		M = 45		0.038 ± 0.119	Nb = 79	0.000	
High age	During	14.08 ± 1.114	FM = 74	-3.159 ± 1.206	0.069 ± 0.104	Nd = 119	0.000	0.131 > 0.05
	After		N = 119		0.072 ± 0.110	Na = 99	0.000	
	Before		M = 57		0.109 ± 0.127	Nb = 93	0.048	
Low myopia	During	12.05 ± 1.903	FM = 74	-2.008 ± 0.674	0.116 ± 0.118	Nd = 131	0.000	0.615 > 0.05
	After		N = 131		0.104 ± 0.117	Na = 92	0.000	
	Before		M = 45		0.070 ± 0.121	Nb = 78	0.000	
Moderate Myopia	During	12.64 ± 1.967	FM = 82	-3.933 ± 0.498	0.094 ± 0.114	Nd = 127	0.000	0.343 > 0.05
	After		N = 127		0.087 ± 0.097	Na = 102	0.189	
	Before				0.094 ± 0.102	Nb = 64	0.002	
Male	During	12.07 ± 2.011	N = 102	-2.752 ± 1.172	0.115 ± 0.115	Nd = 102	0.000	0.865 > 0.05
	After				0.105 ± 0.094	Na = 73	0.029	
	Before				0.084 ± 0.138	Nb = 107	0.000	
Female	During	12.52 ± 1.899	N = 156	-3.089 ± 1.087	0.097 ± 0.117	Nd = 156	0.000	0.448 > 0.05
	After				0.089 ± 0.114	Na = 121	0.000	

^*^Normality test > 0.05, Using one-way ANOVA; Normality test < 0.05, using Kruskal–Wallis H test

*M* stands for male, *FM* stands for female, and *N* represents the total number of subjects, *Nb* represents the number of subjects who had AL change data before the epidemic, *Nd* represents the number of subjects who had AL change data during the epidemic, *Na* represents the number of subjects who had AL change data after the epidemic

This study included 107 subjects who had AL change data before, during, and after the epidemic at the same time. The Repeated-measures ANOVA was done, and *P* (time) > 0.05 was discovered, implying that the AL change did not change with time, and the same result was found in each subgroup. We subsequently discovered that *P* (time*period) for each subgroup was more than 0.05, implying that the magnitude of the change in AL change over time did not differ by gender, age, or initial refraction. Perhaps the only result of concern is that the Test of Between-subjects Effects revealed that age was indeed a significant factor influencing orthokeratology for myopia control in adolescents (Table 3).

**Table 3:** Repeated-measures ANOVA

Group	MTS	TWSE		TBSE	Multiple comparisons between groups		
		P(time)	P(time*period)		Before	During	After
All	0.851	0.082					
Age Group	0.763	0.132	0.051	0.000	0.000	0.001	0.128
Myopia Group	0.839	0.078	0.238	0.999	0.553	0.172	0.590
Gender Group	0.852	0.111	0.747	0.682	0.638	0.771	0.551

Mauchly's test of sphericity were greater than 0.05, so Test of Within-subjects Effects was chosen

MTS represents Mauchly's test of sphericity in our study

TWSE represents Test of Within-subjects Effects in our study

TBSE represents Test of Between-subjects Effects in our study

**Fig. 3:**
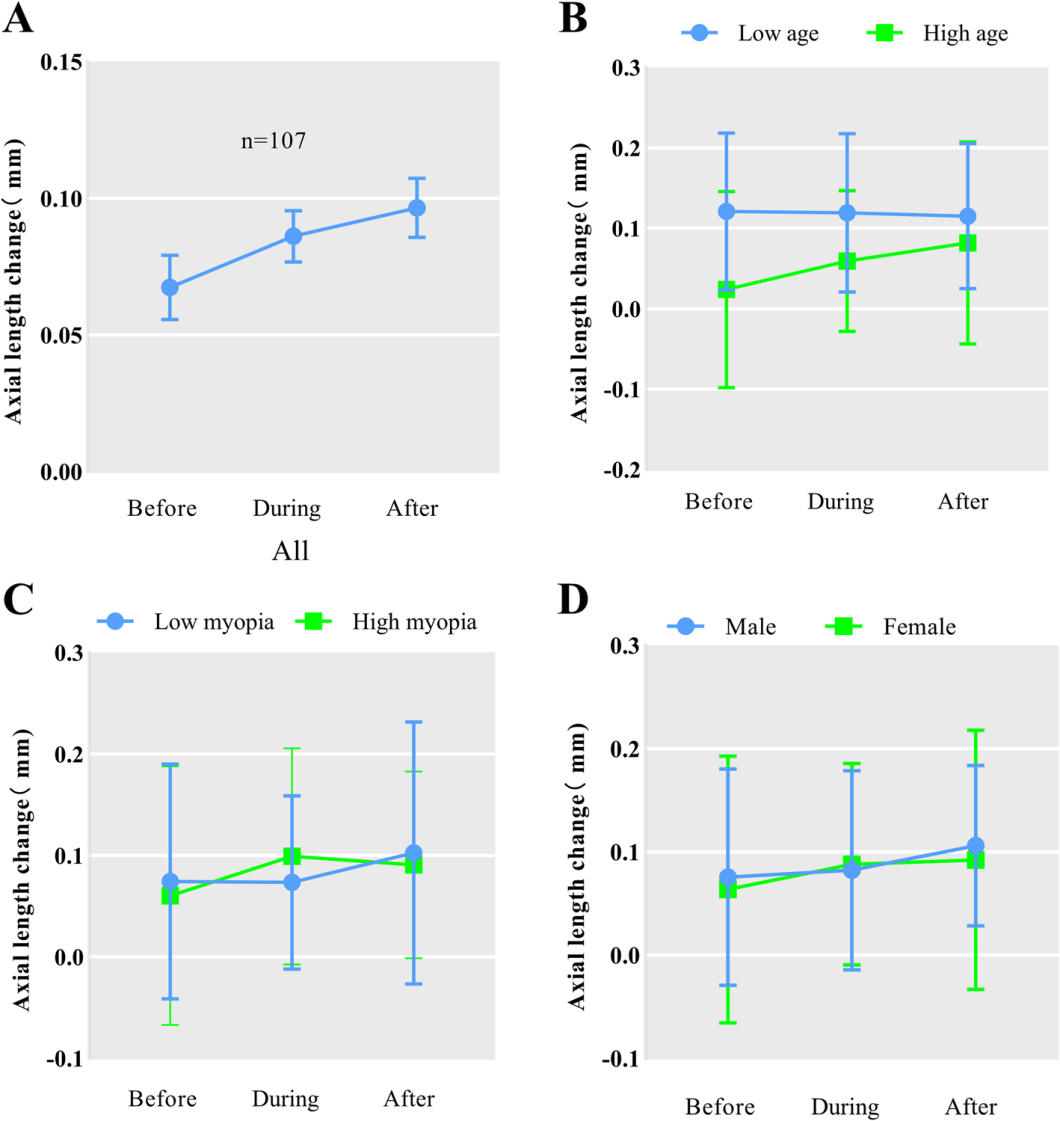
Line chart

Although few statistically significant results were found, we discovered that real situation seems to be different from the statistical results, as we can see from Fig. 3 that there is a linear increase in the AL change over time. Therefore, we conducted a further study with multiple comparisons for each subgroup, and the statistical results are shown in Table 3 and Table 4, which will be used as a reference in the follow-up study.

**Table 4:** The follow-up study of Repeated-measures ANOVA

Group	period	AL change,mm	N	Multiple comparisons within groups	
All	Before	0.067 ± 0.121	*N* = 107	B-D	0.148
	During	0.086 ± 0.097		D-A	0.440
	After	0.097 ± 0.112		B-A	0.028
Low age	Before	0.121 ± 0.098	*N* = 48	B-D	0.924
	During	0.119 ± 0.098		D-A	0.839
	After	0.115 ± 0.090		B-A	0.756
High age	Before	0.024 ± 0.122	*N* = 59	B-D	0.042
	During	0.059 ± 0.087		D-A	0.216
	After	0.082 ± 0.126		B-A	0.001
Low myopia	Before	0.074 ± 0.116	*N* = 54	B-D	0.959
	During	0.073 ± 0.085		D-A	0.130
	After	0.103 ± 0.129		B-A	0.120
Moderate myopia	Before	0.060 ± 0.127	*N* = 53	B-D	0.035
	During	0.099 ± 0.106		D-A	0.666
	After	0.091 ± 0.092		B-A	0.106
Male	Before	0.075 ± 0.105	*N* = 35	B-D	0.763
	During	0.082 ± 0.096		D-A	0.314
	After	0.106 ± 0.078		B-A	0.184
Female	Before	0.063 ± 0.129	*N* = 72	B-D	0.121
	During	0.088 ± 0.098		D-A	0.800
	After	0.092 ± 0.125		B-A	0.076

## Percentage of myopia growth in each group

**Fig. 4:**
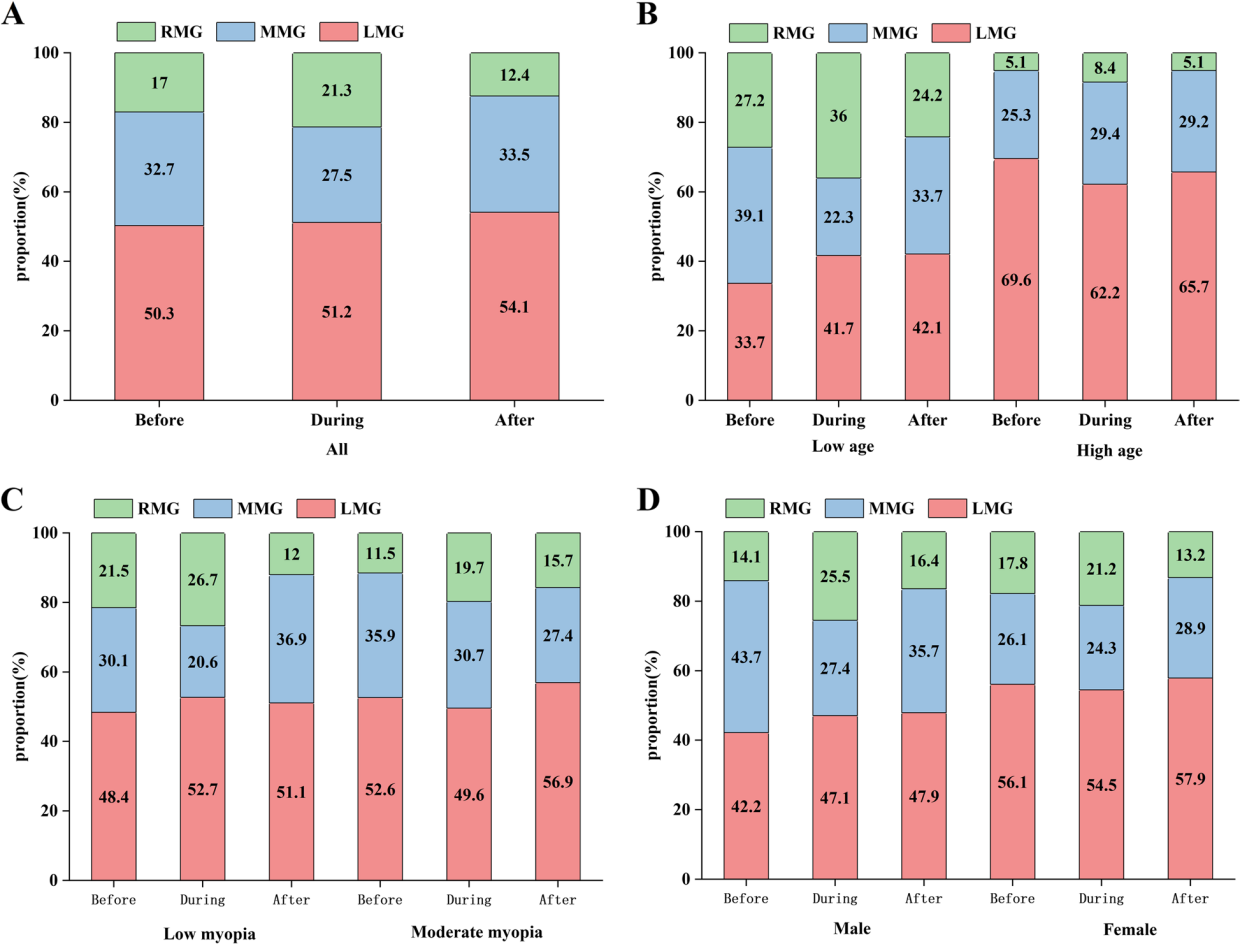
Proportion chart of myopia progression

We calculated the percentage of slow, moderate, and rapid myopia growth in each group before, during, and after the epidemic. Finally, we found that the percentage of rapid myopia growth in the total data and each subgroup increased to varying degrees during the epidemic (Fig. 4). This led us to speculate that home confinement during the epidemic would affect the effectiveness of myopia control with orthokeratology and that this finding differed from the statistical results obtained above.

In addition, we can easily find that the percentage of myopia growth is very different for the low and high age groups, with the percentage of slow myopia growth significantly higher and the percentage of rapid myopia growth significantly lower in the high age group (Fig. 4). We know from this that age can influence the effect of orthokeratology on myopia control, and this variability in myopia control effects by age was also found in the Repeated-measures ANOVA described above. The divergent and linked results obtained above prompted us to change our plan and decide to continue the statistical analysis of the data using paired t-tests and the Wilcoxon signed-rank test in the hope of discovering more accurate results.

In the total data, 171 subjects had AL change data on both before and during the epidemic, with the mean difference ± SD being -0.018 ± 0.133 mm, which were found to be statistically significant using the Wilcoxon signed-rank test (*P* = 0.041). In the high age group, 79 subjects had data on both before and during the epidemic AL change, with a mean difference ± SD being -0.027 ± 0.153 mm, which were found to be statistically significant using the paired t-test (*P* = 0.033). 59 subjects had data on both before and after the epidemic AL change, with a mean difference ± SD being -0.058 ± 0.155 mm, and the Wilcoxon signed-rank test revealed a statistical difference (*P* = 0.023). In the moderate myopia group, 78 subjects had data both before and during the epidemic AL change, with a mean difference ± SD being -0.026 ± 0.152 mm, and a statistical difference was found using the Wilcoxon signed-rank test (*P* = 0.035). In addition, we did not find statistically significant results from other groups (Table 5).

**Table 5:** The difference between AL change of different period

Group	Period	N	Mean difference ± SD, mm	Normality test	p
ALL	b-d	171	-0.0183 ± 0.133	0.000 < 0.05	0.041 < 0.05
	d-a	194	-0.004 ± 0.124	0.000 < 0.05	0.997 > 0.05
	b-a	107	-0.029 ± 0.135	0.000 < 0.05	0.137 > 0.05
Low age	b-d	92	-0.011 ± 0.113	0.310 > 0.05	0.355 > 0.05
	d-a	95	-0.003 ± 0.116	0.384 > 0.05	0.801 > 0.05
	b-a	48	0.006 ± 0.096	0.012 < 0.05	0.693 > 0.05
High age	b-d	79	-0.027 ± 0.153	0.000 < 0.05	0.033 < 0.05
	d-a	99	-0.005 ± 0.131	0.000 < 0.05	0.789 > 0.05
	b-a	59	-0.058 ± 0.155	0.000 < 0.05	0.023 < 0.05
Low myopia	b-d	93	-0.012 ± 0.115	0.206 > 0.05	0.325 > 0.05
	d-a	92	-0.018 ± 0.142	0.000 < 0.05	0.557 > 0.05
	b-a	54	-0.028 ± 0.145	0.000 < 0.05	0.496 > 0.05
Moderate myopia	b-d	78	-0.026 ± 0.152	0.000 < 0.05	0.035 < 0.05
	d-a	102	0.008 ± 0.104	0.104 > 0.05	0.424 > 0.05
	b-a	53	-0.031 ± 0.126	0.000 < 0.05	0.127 > 0.05
Male	b-d	64	-0.027 ± 0.108	0.092 > 0.05	0.050 > 0.05
	d-a	73	-0.012 ± 0.105	0.001 < 0.05	0.704 > 0.05
	b-a	35	-0.031 ± 0.096	0.002 < 0.05	0.154 > 0.05
Female	b-d	107	-0.015 ± 0.146	0.000 < 0.05	0.177 > 0.05
	d-a	121	0.000 ± 0.134	0.000 < 0.05	0.744 > 0.05
	b-a	72	-0.029 ± 0.151	0.000 < 0.05	0.349 > 0.05

^*^Normality test > 0.05, using paired t-test; Normality test < 0.05, using Wilcoxon signed rank test

## Discussion

Myopia has become a worldwide problem that not only poses a great danger to humans but also imposes a significant economic burden on society [39, 40]. The WTO predicts that by 2050, 4.758 billion people (49.8% of the world's population) will be myopic worldwide, and 938 million people (9.8%) are expected to have high myopia (myopia more than -5.00 D) [41]. It is noteworthy that studies have found home confinement affects children's behavioral patterns, such as physical activity (PA) and sedentary behavior (SB), which are associated with physical and mental health, more than we thought [21, 23].  In several subsequent study-based studies, the prevalence of myopia and myopia progression increased significantly during home confinement in children [24–26].

In our study, axial length data in children who treated with orthokeratology were collected retrospectively and prospectively. We performed the Kruskal–Wallis H test and the Repeated-measures ANOVA on the total data and each subgroup. No statistical significance was found between the AL change before, during, and after the epidemic. (Fig. 2) Based on this, we may tentatively conclude that the epidemic did not have an impact on the use of orthokeratology to control myopia and seem to have reached the same conclusion as previous studies [42].

However, we also observed some interesting characteristic changes during home confinement, such as an increase in the percentage of patients with rapid progression compared to before and after confinement. (Fig. 4) This characteristic change suggests a definite effect of home confinement during the epidemic, which was contradictory to the findings presented above. It is important to note that most children in urban have a poorly natural light exposure environment compared to classroom, and numerous studies have demonstrated the association between light and myopia [43, 44]. The second is that during home confinement, children have to be educated online, and activities performed in front of a digital screen may have a different effect on myopia than reading and writing in traditional education [45, 46]. Prolonged close use of electronics is also known to affect myopia progression [47, 48].  These factors suggest that myopia control with orthokeratology may be compromised during the epidemic. However, this study did not get a definite statistical result. We hypothesize the reason why is that some children with orthokeratology were significantly affected by home confinement, while most were only marginally affected, just because of the different compliance, and the irregularity of work and rest leading to insufficient OK lens wear time. However, most patients do not remember the duration of OK lens wear and outdoor activities, some patients exaggerate the duration of OK lens wear. This factor relies entirely on patient subjectivity and may produce misleading results in our study, so we did not consider it. Nevertheless, in other prospective studies where the authenticity of the data can be controlled, patient compliance is an important factor which needs to be taken into consideration.

The Repeated-measures ANOVA is theoretically the most suitable statistical method for this study. But we found from Fig. 3 that the real situation does not seem to be as expressed by the statistical results. In addition, the sample size suitable for repeated measures ANOVA was small, so we used the collected data for a paired t-test as a reference. Although they did not take time into account, but we need to note that a study has found that the effect of myopia control by orthokeratology only diminished statistically in the fourth year, [28] and the process of orthokeratology wear was supervised by professional doctors and optometrists to minimizes the impact of time. Therefore, it is believed in our study that there is some justification for using paired t-tests and Wilcoxon signed-rank tests.

**Fig. 5:**
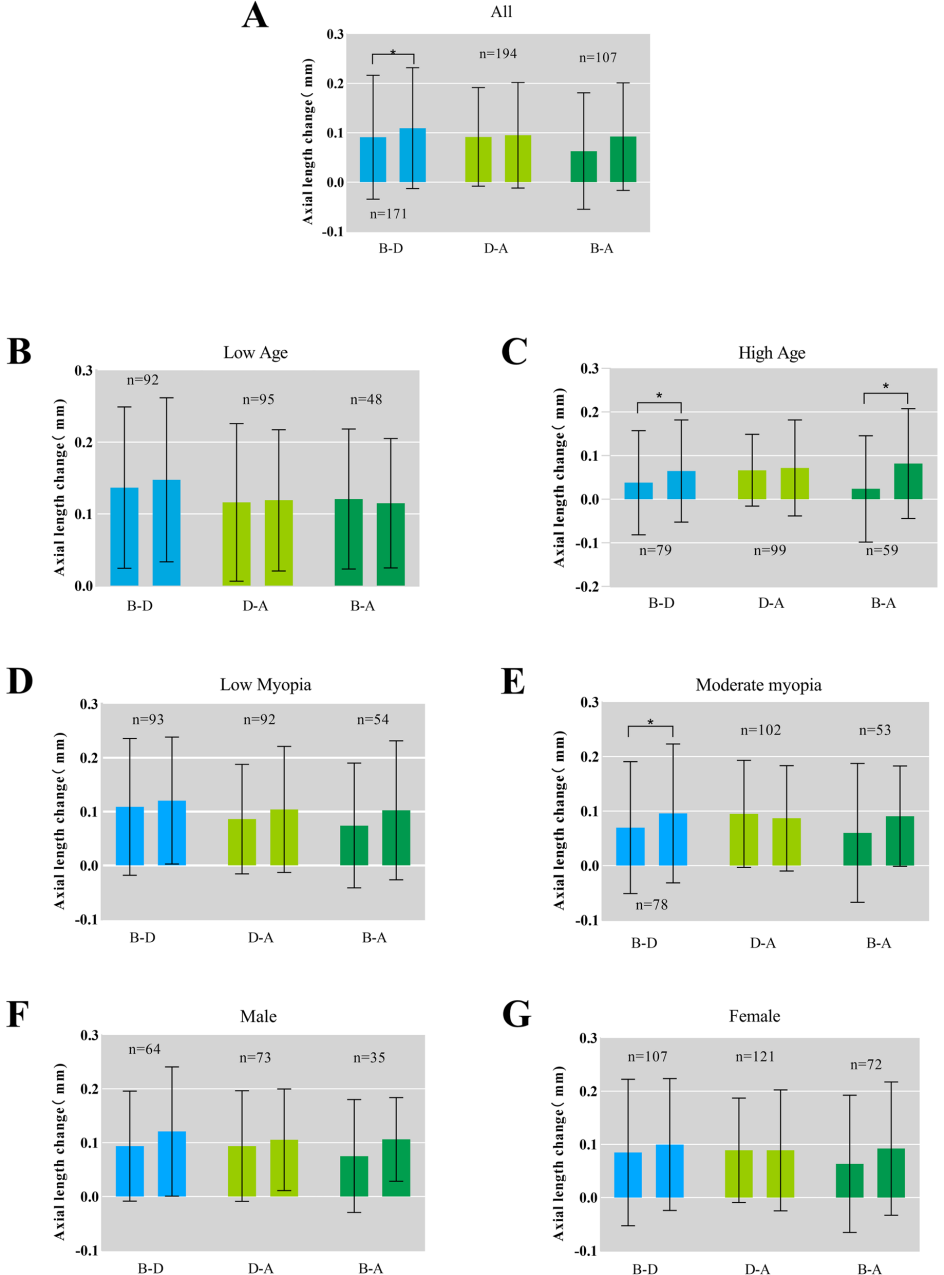
Box plot

When we used paired t-test in the total data, we found a statistically significant difference between the AL change before and during the epidemic, (*n* = 171) and from Fig. 5(A), we also found that the AL change during the epidemic was faster than before the epidemic, which shows that the effect of orthokeratology on myopia control was reduced during the home confinement. Subsequently, we conducted statistical analyses the AL change during and after the epidemic, (*n* = 194) before and after the epidemic, (*n* = 107) and found no statistical results, which leads us to speculate that the impact did not fully return to the level before the epidemic. Even it is more far-reaching than we thought. The opposite results obtained using this statistical method, in our opinion, are mainly due to the difference in sample size. However, it is not known whether there will be significant results when the sample size of repeated measures ANOVA is sufficient, and data integration for large sample analysis or Meta-analysis is necessary when the number of relevant studies is enough.

When we performed the Wilcoxon signed-rank test and paired t-test on the moderate myopia group, we found significant differences between the AL change before the epidemic and during the epidemic (*n* = 78). And there were no significant differences between the AL change before and after, (*n* = 102) during and after the epidemic (*n* = 53). In contrast, no significant differences were obtained for the low myopia group. Comparing the two with each other, we assume that the children with moderate initial refraction were more likely to be affected by the home confinement. In addition, no significant differences were obtained in gender groups, so we conclude that the gender differences were equally affected by home confinement.

Finally, from Fig. 4(B) we found that younger children have a smaller percentage of slow myopic growth and a larger percentage of rapid myopic growth compared to older, indicating that age is a factor that impacts the efficiency of orthokeratology. We then found significant differences between the AL change before and during, (*n* = 79) before and after the epidemic in high age group (*n* = 59). In contrast, there were no differential results for the low age group. Therefore, we concluded that the older children were more susceptible to home confinement for myopia control with orthokeratology. We guess the possible reasons is that older children have more stressful schoolwork. When more time is spent using electronics during home confinement, the corresponding time spent using OK treatment may be reduced.

An interesting phenomenon is that when we look back at the results of the repeated measures ANOVA multiple comparisons (Table 4), we found that the subgroups obtained the same differential results as using the paired t-test and Wilcoxon signed-rank test. We found from Fig. 3 that the real situation does not seem to be as expressed by the statistical results. There seems to be a changing curve in the total sample (*n* = 107). These connections and differences allow us to venture a guess that the results of the repeated measures ANOVA obtained in our study are opposite to the actual, owing to the small sample size.

Studies have shown that myopia progression in Chinese children is related to the seasons [49]. The AL changes are larger in the autumn and winter compared to the spring and summer. And this seems to better support the conjecture of our study. The periods were chosen as close to a six-month interval as possible, whereas the epidemic period had to encompass February through May, so that spring and summer dominated the epidemic period, while the pre-epidemic and post-epidemic periods were more autumn and winter, so theoretically this study should have shown smaller AL change and correspondingly better myopia control during the epidemic. But in fact, we found that most of the significant results showed larger AL changes during the confinement, which contrary to the previous study. Therefore, if the impact of seasons on children's myopia control is taken into account, we should consider that the effect of home confinement may be more powerful than we thought.

Although we found a lot of interesting characteristic changes and made speculations based on that, this study still has some problems include the lack of randomization, insufficient sample size, and the fact that further research is needed to taking patient compliance into account, so we cannot conclude that home confinement did have a negative impact. Additionally, it is difficult to reproduce such a study because of the unpredictability of large-scale epidemic home confinement measures. Follow-up observations of junior and senior students using orthokeratology could be considered to improve this study.

## Conclusion

As a result, we cannot conclude that home confinement did have a negative impact on myopia control with orthokeratology in school-aged children, orthokeratology is still an effective means for controlling myopia in children. But we found some characteristic changes. There was an increase in the percentage of patients with OK treatment that had fast myopia progression during the confinement. We also observed that older children with higher initial refraction were more likely to be affected by home confinement. Our study will provide a basis for future policy development, and some findings of this study will also recommend that health care providers give more attention to older children with high refraction, especially in those children who may have experienced change in behavioral patterns, such as reduced physical activity (PA) and prolonged sedentary behavior (SB), similar to the epidemic home confinement situation.

## Supplementary Information

**Additional file 1.** 

**Additional file 2.** 

**Additional file 3.** 

## Data Availability

The data used to support the findings of this study are available from the corresponding author upon request.

## Abbreviations

AL: Axial length
PA: Physical activity
SB: Sedentary behavior
COVID-19: Corona Virus Disease 2019
IOL master: Intraoeular len master
CA: California
Spss: Statistical Product Service Solutions
IL: Illinois
ANOVA: Analysis of Variance
SD: Standard Deviation
